# Gastric Outlet Obstruction Caused by Adhesion Between Remnant Greater Omentum and Lesser Omentum After Laparoscopic Transverse Colectomy

**DOI:** 10.1111/ases.70249

**Published:** 2026-02-05

**Authors:** Kyosuke Yoshitake, Jun Nagata, Hiroto Sannomiya, Kensuke Nitta, Atsuhiro Koga, Yasuki Akiyama, Yasuhisa Mori, Masumi Yamauchi, Nagahiro Sato, Toshihisa Tamura, Kazunori Shibao, Keiji Hirata

**Affiliations:** ^1^ Department of Surgery 1 University of Occupational and Environmental Health Kitakyushu Fukuoka Japan

**Keywords:** adhesion, gastric outlet obstruction, greater omentum, laparoscopic colectomy, lesser omentum

## Abstract

Postoperative adhesive small bowel obstruction is common after abdominal surgery, whereas gastric outlet obstruction (GOO) due to adhesions on the anterior surface of the stomach is extremely rare. We report an 81‐year‐old man who developed persistent vomiting shortly after laparoscopic transverse colectomy. Computed tomography revealed marked gastric dilatation with a beak sign at the pylorus, and upper gastrointestinal endoscopy showed stenosis from the lower gastric body to the gastric angle without intraluminal lesions. Emergency laparoscopy identified a band‐like adhesion between the lesser omentum and the remnant greater omentum crossing the anterior wall of the pyloric region and constricting the gastric outlet. Laparoscopic adhesiolysis with intraoperative endoscopic confirmation of patency relieved the obstruction, and the patient recovered uneventfully. This case highlights that omental–lesser omental bands should be considered as a differential diagnosis of early postoperative GOO after colorectal surgery and that laparoscopic adhesiolysis is a safe, minimally invasive treatment option.

## 1 Introduction

Postoperative adhesions are among the most frequent complications after abdominal surgery and are a leading cause of small bowel obstruction. In contrast, gastric outlet obstruction (GOO) due to adhesions on the anterior surface of the stomach is extremely rare. Common causes of GOO in adults include malignancy of the distal stomach and cicatrized duodenal ulcer, whereas benign mechanical obstruction related to peritoneal bands or omental adhesions is uncommon.

Peritoneal bands can arise congenitally or be acquired after inflammation or surgery, and they are well known as a cause of small bowel obstruction. Recently, an anatomical case describing constriction of the stomach by an unusual peritoneal band has been reported, suggesting that peritoneal folds can occasionally compromise gastric patency. However, postoperative GOO caused by an adhesion between the remnant greater omentum and the lesser omentum after colorectal surgery has rarely been described.

We present a case of GOO due to a band‐like adhesion between the remnant greater omentum and the lesser omentum following laparoscopic transverse colectomy, successfully treated by laparoscopic adhesiolysis. We also discuss possible mechanisms of band formation and strategies for prevention.

## 2 Case Presentation

An 81‐year‐old man underwent laparoscopic partial transverse colectomy with D3 lymphadenectomy for transverse colon cancer. The greater omentum was divided using ultrasonic shears along the gastrocolic ligament toward the hepatic flexure. The hepatic flexure was mobilized, while the splenic flexure was not mobilized. The anastomosis was performed extracorporeally via a 4‐cm supraumbilical mini‐laparotomy using functional end‐to‐end stapling. The divided omental edge was left in the upper abdomen without fixation. No congenital or pre‐existing adhesions between the greater and lesser omentum were observed. An adhesion barrier (Seprafilm) was applied to the mesocolic defect and lymphadenectomy bed but not to the divided omental edge. Operative time was 248 min, and blood loss was 20 mL.

The postoperative course was unremarkable except for a single episode of non‐bilious vomiting on POD 13, which resolved spontaneously without abdominal pain or distention. No imaging was performed, and oral intake resumed. The patient was discharged on POD 22.

**FIGURE 1:**
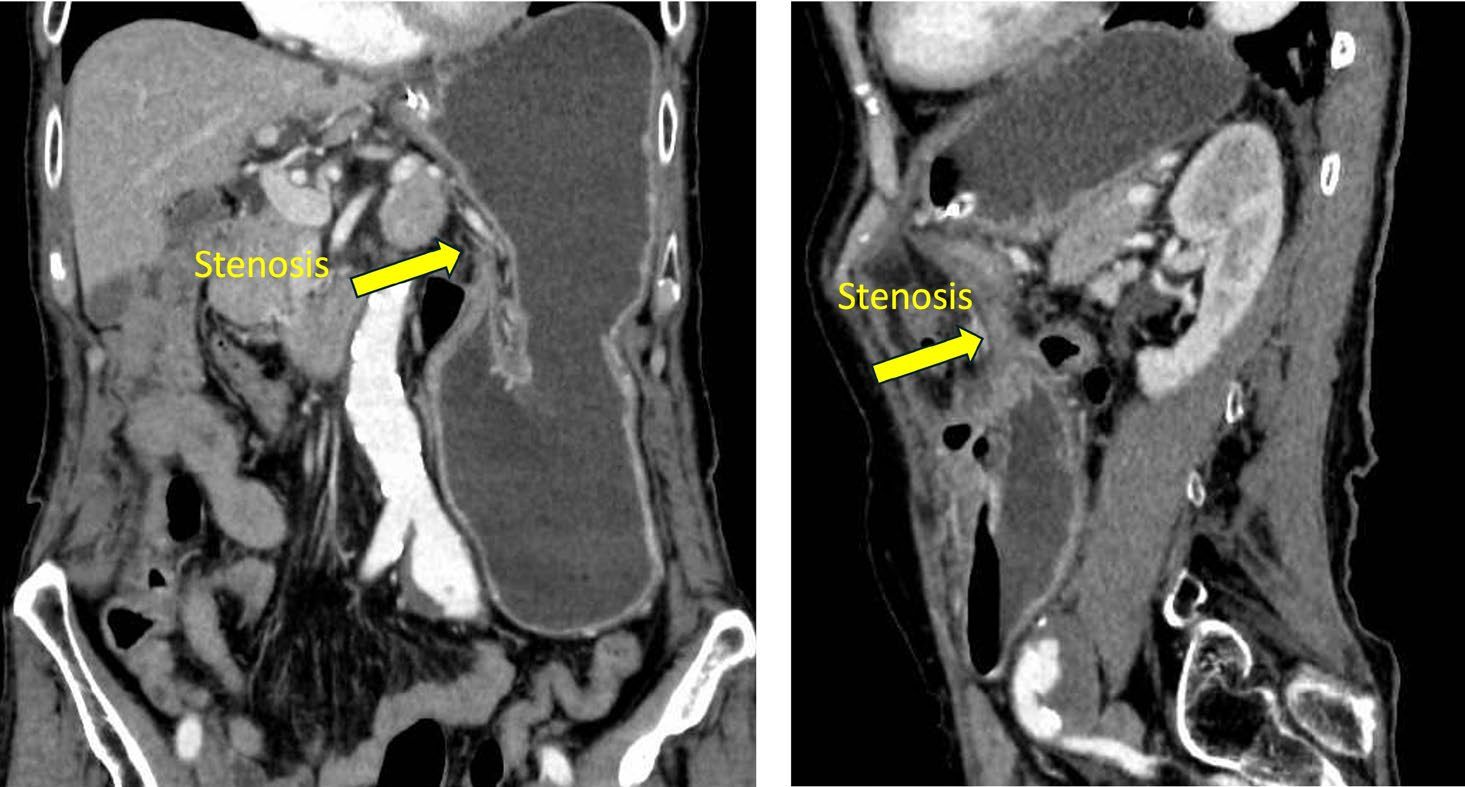
Contrast‐enhanced computed tomography showing marked gastric dilatation and a beak sign at the pyloric region, indicating a transition point of obstruction without small bowel dilatation.

Two days after discharge, he developed persistent vomiting and was readmitted. His abdomen was soft and non‐distended. Laboratory tests were normal. Contrast‐enhanced CT demonstrated marked gastric dilatation and a beak‐like narrowing at the pylorus (Figure 1). Upper gastrointestinal endoscopy revealed severe stenosis from the lower gastric body to the gastric angle; the endoscope could not be advanced.

**FIGURE 2:**
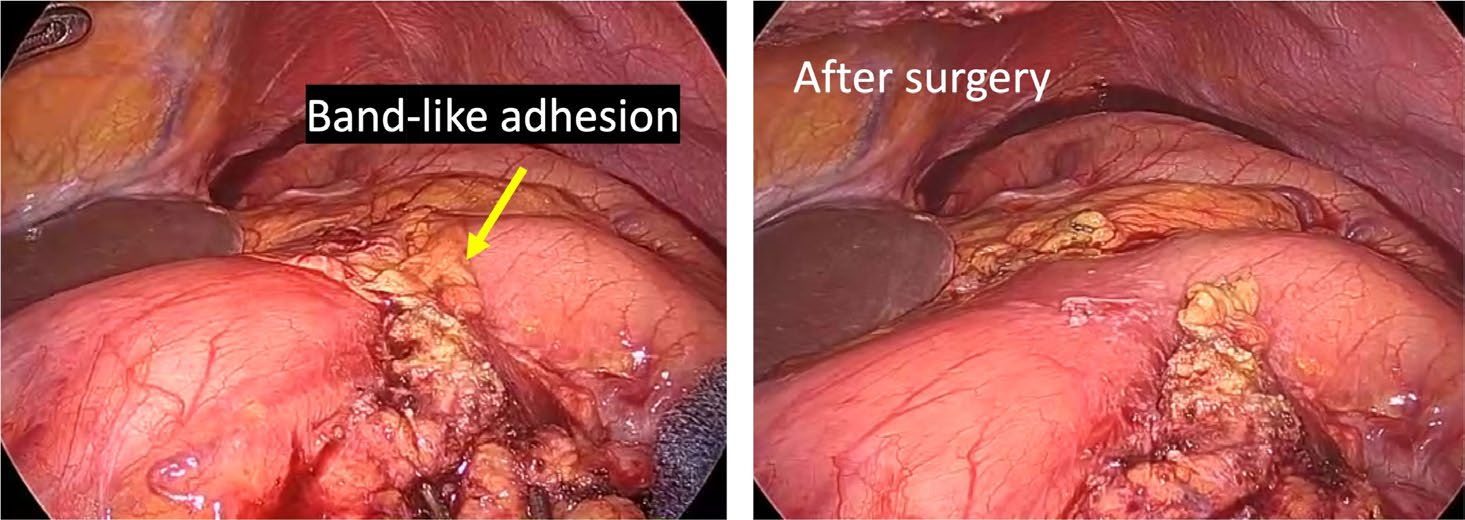
Laparoscopic view of a band‐like adhesion between the lesser omentum and the remnant greater omentum crossing the anterior wall of the pyloric region and constricting the gastric outlet [left panel]. After resection of a band‐like adhesion [right panel].

Emergency diagnostic laparoscopy was performed on readmission day 2 (POD 24). Operative time was 94 min, and blood loss was < 5 mL. A band‐like adhesion connecting the lesser omentum and the divided greater omentum was identified, compressing the anterior wall of the pyloric region (Figure 2). Laparoscopic adhesiolysis restored luminal patency. Intraoperative endoscopy confirmed smooth passage into the duodenum. The patient resumed oral intake and was discharged without recurrence.

This complication corresponds to Clavien–Dindo grade IIIb.

## 3 Discussion

This case illustrates an exceptionally rare mechanism of postoperative GOO: a band‐like adhesion between the remnant greater omentum and the lesser omentum compressing the anterior pyloric region after laparoscopic transverse colectomy. Although adhesive small bowel obstruction is relatively common after colorectal surgery, adhesion‐related GOO is exceedingly uncommon. A limited number of reports have described similar mechanisms. Chen et al. reported a case of acute GOO after left hemicolectomy caused by an adhesive band extending from the antrum to the abdominal wall [1], demonstrating that postoperative adhesions following colorectal procedures can obstruct the gastric outlet. Kassem et al. also described constriction of the stomach by an unusual peritoneal band [2], highlighting that aberrant peritoneal adhesions may compromise gastric patency even in anatomically unexpected sites. However, to our knowledge, no previous report has documented GOO caused specifically by adhesion formation between the divided greater omentum and the lesser omentum, underscoring the novelty of the present case.

The mechanism in our patient likely involved secondary adhesion between the raw cut surface of the greater omentum and the lesser omentum within the upper abdomen, forming a tension band across the anterior pylorus. Because the greater omentum is highly mobile, partially divided segments may migrate and attach to adjacent organs during postoperative inflammation and fibrosis. In this case, the resulting adhesion progressively constricted the pyloric region.

When early postoperative vomiting occurs without intraluminal pathology, adhesion‐related GOO should be considered as a differential diagnosis, even after colorectal surgery. Cross‐sectional imaging and upper gastrointestinal endoscopy are valuable for identifying the level of obstruction. Diagnostic laparoscopy, as demonstrated in this case, enables direct visualization of atypical adhesive bands and allows definitive treatment through safe adhesiolysis. Intraoperative endoscopy contributed by confirming restoration of luminal patency after division of the adhesion.

This case also raises important technical considerations related to omental handling. Raw or irregular omental edges left adjacent to the stomach may serve as a nidus for unexpected adhesions. Preventive strategies may include careful trimming of the divided omentum, ensuring that its final position is away from the gastric outlet, and considering the application of adhesion barriers when feasible, although evidence remains limited.

In summary, postoperative GOO caused by adhesion between the remnant greater omentum and the lesser omentum is extremely rare. Clinicians should be aware of this unusual mechanism, and diagnostic laparoscopy is a reliable method for both diagnosis and treatment.

## 4 Conclusion

GOO caused by adhesion between the remnant greater omentum and the lesser omentum is an exceedingly rare postoperative complication following colorectal surgery. It should be considered when early postoperative vomiting with gastric dilatation occurs. Diagnostic laparoscopy provides both definitive diagnosis and effective treatment.

## Funding

The authors have nothing to report.

## Ethics Statement

According to the institutional policy for single case reports, formal ethics committee approval was waived.

## Consent

Written informed consent for publication of this case and accompanying images was obtained from the patient.

## Conflicts of Interest

The authors declare no conflicts of interest.

## Data Availability

Data sharing is not applicable to this article as no datasets were generated or analyzed during the current study.
